# Understanding social support during pregnancy: A qualitative study of women’s lived experiences in Nepal

**DOI:** 10.1371/journal.pone.0333885

**Published:** 2025-10-17

**Authors:** Lalita Kumari Sah, Eleni Hatzidimitriadou, Rajeeb Kumar Sah

**Affiliations:** 1 Faculty of Health Studies, University of Bradford, Bradford, United Kingdom; 2 Faculty of Medicine, Health and Social Care, Canterbury Christ Church University, Canterbury, United Kingdom; 3 Human and Health Sciences, University of Huddersfield, Huddersfield, United Kingdom; Menzies School of Health Research: Charles Darwin University, AUSTRALIA

## Abstract

This research study examines the experience and understanding of available social support in Nepal and its importance during pregnancy. The interviews, utilising twenty in-depth interviews with pregnant women in Nepal, were analysed inductively using a thematic analysis approach. The findings suggested that women consider their husband’s support the most important and desired support during pregnancy, while women also acknowledged support from relatives and neighbours. None of the women experienced any organised community support or NGO/INGO support, and the support from healthcare providers was limited. At the policy level, the maternity leave policy by the government of Nepal is seen as inadequate support in promoting working women in Nepal, and it lacks implementation in informal work sectors. The study concludes that appropriate social support can significantly enhance the well-being of pregnant women. Given the limited availability of formal support systems and the central role of spousal and informal community support, these findings highlight the need for context-specific interventions. Therefore, we recommend conducting interventional studies to design and evaluate targeted support mechanisms that address the diverse needs of pregnant women in Nepal, shaped by their socioeconomic and cultural contexts.

## Introduction

Social support is essential during pregnancy since support received by pregnant women has a positive impact on newborn babies and new mothers, as well as the family [1]. Experience of social support remains subjective and dependent on the perception of individuals. However, in general agreement, social support is any form of social interaction that supports the well-being of the recipient of support [2,3]. During pregnancy, many women experience significant changes in terms of physical, emotional and psychological well-being, and it can be a challenging experience in many cases. Adequate support in any form, such as emotional, informational or instrumental, has been seen to make a positive impact as it supports coping with the challenging experience during pregnancy and improves health outcomes [4]. On the other hand, low social support may put pregnant women at significant risk of poor health and well-being, such as depression, anxiety, and self-harm [5,6]. Social support can be received from different sources, such as husband/partner, family/relatives, neighbours, and community, as well as from the nation’s welfare system, which we explore in this paper in the context of pregnant women in Nepal.

Few studies conducted previously in South Asia found that adverse social environments and social relations have a significant adverse impact on health of pregnant women, such as women experiencing any form of violence, conflict situations in the family and communities, exposure to natural disasters, and low-level social support are major determinants of poor health for women during pregnancy [7–9]. Within the wider South Asian context, these findings may suggest similar hidden concerns regarding the mental health of pregnant women in Nepal. However, there is a dearth of research that primarily explores the social support for pregnant women in the South Asian context, including Nepal. In Nepal, few research studies are focused on antenatal services [10,11], but hardly any research directly focuses on exploring the experience and understanding of social support of pregnant women. Therefore, this paper, the first to our knowledge, aimed to examine women’s experience and understanding of social support during their pregnancy.

### Context of pregnancy and social support

Pregnancy is a time when women experience several physical, social and emotional changes. The hormonal changes during pregnancy may cause physical and emotional changes, and certainly, some changes may put them at risk of vulnerability to poor mental health [12,13]. In addition, many women may experience changes in their roles and responsibilities towards society and family, alongside financial burdens associated with pregnancy and birth [14–16]. During this motherhood transition, pregnant women generally receive support from close family members, such as parents and in-laws in Nepal [17]. However, this may not be the case for all pregnant women due to several socioeconomic and cultural circumstances. Evidence states that the lack of social support and the poor relationship between pregnant women and their family members, such as husbands and in-laws, is associated with poor mental health experiences during pregnancy [18,19].

Social support can be emotional, informational or instrumental [20]. Previous research has identified that the most needed support during the maternity period is emotional and informational [21]. Social support also plays an important role in promoting mental health and well-being, and adequate social support is essential to cope with adverse and stressful situations [22] but there is very limited research about the wider social support for Nepalese women during their pregnancy. Generally, women with a lower socioeconomic position face more challenges [23], such as social pressure to maintain social expectations from social connections, that put them at risk of poor mental health [24,25]. Therefore, risks and benefits should be analysed before promoting social support interventions. Considering such arguments, this paper aims to examine the experience and understanding of social support through the lived experience of pregnant women in Nepal.

### Ecological theory and intersectionality in the context of social support

Utilising Bronfenbrenner’s Ecological Theory [26], this research aims to examine the experience and understanding of social support of pregnant women within the multiple layers of the environment they live in. The pregnant women positioned at the centre of the ecology may experience support from immediate family to broader social and policy level contexts, where they have little or no control to influence these contexts in their favour. Looking at the microsystem level, the most immediate environment for the women includes family members, husbands/spouses, neighbours and healthcare providers who can have a significant influence and provide support during the pregnancy. The relationship of women within the immediate environment could be a critical source of emotional, informational or tangible support, such as helping with household tasks. However, given the extended family structure, generational expectations and patriarchal norms, the immediate environment may cause conflict and contribute to stress due to the restricted role of the daughter-in-law [27]. Within the exosystem of the ecology, we can expect that local government and municipalities, grassroots health programmes, and local non-government organisations (NGOS) can potentially provide support and assistance to pregnant women that directly influence the overall experience and outcomes of maternal health and wellbeing. However, these support may depend on the resources available at the local level and may vary in each community. Within the macrosystem of ecology, we understand that women have no influence or control over the resources and policies related to maternal health and social protection that are at the national level and sometimes influenced by international commitments of the government, such as achieving sustainable development goals (SDGS). International non-government organisations (INGOS) also play key roles in facilitating support for pregnant women by influencing policy and programmes at the national level.

To broaden our understanding of the context of social support at multiple levels influenced by different identities of women, we also apply the concept of intersectionality [28]. This concept allows us to see how multiple identities of the women, such as age, geographical location, education, and economic status, overlap and influence the social support they need and receive during their pregnancy. For example, pregnant women living in rural areas may have limited household income and education, which may limit their ability to utilise the existing social support in the community and nationwide due to limited awareness and accessibility to resources. These factors can create compounded risk to many women, putting them at risk of vulnerability to timely access to health services and social support, leading them to experience stress and anxiety about their unborn baby and themselves. The concept of intersectionality allows this research to see the intersection of individual, family and social circumstances, where some women may feel further disadvantaged in accessing the support available to them, affecting their mental health and well-being. By integrating Bronfenbrenner’s ecological theory with the concept of intersectionality, we aim to provide a deep analysis of the experience and understanding of social support of pregnant women in Nepal and its importance in addressing maternal health, by exploring multiple levels of support, which involve individuals, organisational and policy levels.

Based on the discussion so far, this study draws upon Bronfenbrenner’s ecological theory and the concept of intersectionality to explore the complexity of pregnant women’s social support systems in Nepal. While social support is often discussed as a single-layer phenomenon, these frameworks allow us to understand support as multi-layered (individual, community, policy), which is shaped by intersecting identities such as gender, class, rural/urban residency, and marital status. Together, they inform our understanding of how contextual and structural conditions influence the quality, type, and accessibility of support experienced by pregnant women.

## Research method

In this study, we aim to present the experience and understanding of social support among pregnant women in Nepal, utilising data from in-depth interviews with twenty pregnant women. Given the descriptive and exploratory nature of the study, the qualitative approach in this research enabled us to gather participants’ perceptions and experiences of the support they received during their pregnancy and its impact on them [29–31]. The women were encouraged to share their lived experiences in the form of narration/story, while providing flexibility on how they wanted to respond [32]. The study employed in-depth, open-ended interviews, allowing participants to speak freely about the aspects of their maternal journey that were most important or meaningful to them. While an interview schedule/guide was used to guide the conversation, participants were not pressured to follow a strict sequence or to move on to other sections prematurely. This approach created space for participants to share their personal experiences naturally and flexibly, fostering rich and authentic narratives.

### Data collection

In-depth interviews with 20 pregnant women were conducted by the first author within the context of her PhD project. Data collection was completed at a District Hospital in the Eastern region of the Koshi Province in Nepal. The women were approached when they were waiting to see the doctors or get test results in the waiting area of the maternity unit. The hospital provided access to participants and played the gatekeeper role in this research. Purposive convenience sampling [33] was used as the most suitable approach in this research, as the participants’ convenience was the most important factor. For example, participants’ convenience was based on participants’ availability, willingness to participate, and convenient time. This data was collected virtually due to the COVID-19 pandemic, between September and November 2020, and both verbal and written consent were obtained prior to the interviews.

The average time of in-depth interviews was 30–45 minutes. However, the first author of this paper, who conducted interviews, spent 10–15 minutes with each pregnant woman to build rapport and trust before starting the interviews and after the interview to ensure they were satisfied and confident with the interview process. The inclusion criteria of the participants were:

- a pregnant woman at any stage of their pregnancy (regardless of the history of the number of births and pregnancies)
- 18 years and over
- living in the region and accessing maternal healthcare services at the District Hospital,
- have the ability to give consent to participate in the study

Prior to data collection, two pilot interviews were conducted to avoid technical terminology and confusion in the participant information sheet, consent form, and other documents, as well as to improve the interview strategy, including prompts. The two pilot interviews were not included in the interview number mentioned above. The pilot interviews also helped to develop the interview guide to use in later interviews. In total, twenty interviews were conducted with pregnant women. However, the last three interviews did not introduce any new themes or ideas, which clearly indicated data saturation; therefore, no further interviews with pregnant women were required after the twentieth interview.

All the interviews were audio recorded with consent, and the language used in the interviews was Nepali. Being bilingual in Nepali and English, the first author conducted all the interviews and transcribed all the interviews in the Nepali language and then translated them into English. She, the first author, anonymised the data to maintain the anonymity and confidentiality of the participants. For quality purposes and to ensure the accuracy of the translation, three translated interviews were ‘back translated’ into the Nepali language by a bilingual colleague who knew both English and Nepali, in line with the discussion within the literature [34]. The ethics were obtained for this research from the Nepal Health Research Council (Registration No 356/2020) and the Canterbury Christ Church University, UK (Ref: ETH1920−0026).

The hospital played a key role as a gatekeeper in the recruitment process. Hospital staff identified eligible participants attending the antenatal clinic and provided them with information about the study. Study information leaflets were made available in the clinic area for attendees to read and take voluntarily. Staff also introduced the study to potential participants and asked if they were interested in taking part. Women who expressed interest were given sufficient time to read the participant information sheet. Some participants chose to take part on the same day, especially if they were not planning a return visit to the hospital, while others agreed to participate later. Once a participant showed interest, the gatekeeper introduced her to the principal investigator, who explained the study’s purpose, procedures, and ethical safeguards in more detail. Written informed consent was obtained prior to each interview.

Due to practical considerations, all interviews were conducted virtually using audiovisual platforms such as WhatsApp. The hospital provided the phone that was securely kept in the hospital following the hospital’s storage and security policy. The quiet room with a lockable door was allocated for the interview, and no one was present in the room at the time of the interview except the participant. While video was used to establish rapport, only the audio portion of each interview was recorded for transcription and analysis purposes. Participants completed and signed consent forms with support from the researcher, who clarified any questions the participants had. Signed forms were scanned and submitted electronically via a secure platform. In accordance with hospital policy and ethical approval conditions, all physical copies of consent forms were destroyed to ensure confidentiality. This process ensured voluntary participation and protected participants’ privacy while maintaining research integrity. Given the constraints imposed by the COVID-19 pandemic, virtual data collection was not a choice, but rather the only option. The broader literature supports the use of virtual interviews in research, noting that there is no significant difference in the depth or quality of data collected compared to in-person methods [35–37]. Drawing on our previous experience conducting qualitative interviews as researchers and supervisors, we were able to apply reflexivity throughout the interview process. This practical experience enhanced our confidence in the effectiveness of virtual interviews, and we found that they yielded rich and meaningful data comparable to that obtained through face-to-face interactions.

### Data analysis and interpretation

Reflexive thematic analysis, as developed by Braun and Clarke [38,39] was used to analyse data using an inductive approach [40]. Thematic network tool [41] and Nvivo12 were used to organise the themes. The thematic network tool helped to organise themes into three levels: basic themes, organising themes and the global theme. While the global theme is ‘social support of pregnant women’, organising themes (subthemes) are: family support; support from relatives, neighbours and community; organised community support; support from NGO/INGO; and maternity leave. The basic themes are presented as quotes and explanation within the findings section. Code-recoding and analysing strategies were applied robustly during data analysis, in which team members checked coding consistency over time. The research project supervisors conducted a peer review to ensure consistency in data interpretation. Peer debriefing and discussing the analysis with team members and stakeholders ensured that this research was challenged to maintain interpretations without bias. The previous experience of the first author, having experience conducting interviews and completing research in the past, is further supported in the reflexivity practice in this research. The researcher and the team of supervisors were self-aware of their position as researchers and supervisors, where their own values and beliefs had no or possibly limited influence in this research, which we acknowledged in the limitations of this research. Reflexivity was central throughout the process, recognising that the researchers’ positionalities, backgrounds, and perspectives inevitably shaped the interpretation of data. Efforts were made to engage critically and transparently with the meanings generated in the analysis. Overall, the first author, being a Nepali woman with prior experience conducting qualitative interviews, brought valuable cultural insight and empathy to the analysis. Her insider status enabled deeper engagement with participants’ narratives, while supervisory peer review and critical discussions helped ensure that interpretations remained grounded in the data and not overly influenced by personal assumptions. This reflexive engagement with theory, data, and interpretation is consistent with Braun and Clarke’s approach to reflexive thematic analysis. Therefore, we justify the use of reflexive thematic analysis (RTA) and application of original work by Braun and Clarke [38,39] to analyse the data. An inductive, data-driven approach guided the development of codes and themes. The six-phase process, as mentioned by Braun and Clarke, involved: 1. familiarising with the data, 2. generating initial codes, 3. searching for themes, 4. Reviewing themes, 5. defining and naming themes, 6. producing tables with extract examples. Throughout the analysis, we adopted a reflexive approach, acknowledging the influence of our own positions and experiences on our interpretation. While coding was not guided by theoretical assumptions, ecological theory and intersectionality were used at the interpretive level to deepen understanding of the findings within their social and structural context.

## Findings

**Fig 1:**
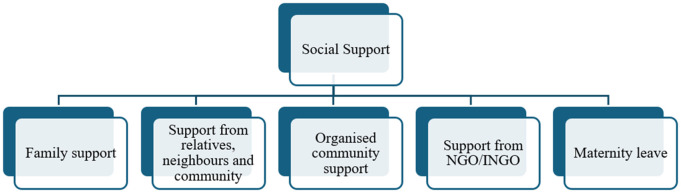
Experience and understanding of social support of pregnant women in Nepal.

Although the coding was conducted inductively, theoretical frameworks: ecological theory and intersectionality, were applied during the interpretive stage to contextualise and enrich the understanding of the emergent themes. Following the concept of thematic network tool presented by Attride-Stirling [41] to organise themes, this findings section presents the experience of social support that has been categorised into five organising themes, and the basic themes are presented within the quotes of the participants. The finding section starts by looking into immediate family support, support from relatives, neighbours and community, organised community support, support from NGO/INGO, and finally the maternity leave policy in Nepal, which determines the extent to which the support system is available for pregnant women in Nepal. Irrelevant conversation has been replaced by […..] to maintain the flow and focus of the quotes presented below. Fig 1 presents a visual representation of the organising themes based on the inductive data analysis used in this study.

### Family support

This theme sits within the microsystem of ecological theory, highlighting how immediate relationships, especially with husbands, shape women’s experiences. Understanding through an intersectional lens, the quality of this support is strongly influenced by socioeconomic status, migration of spouses, and traditional gender expectations, leading to unequal experiences even within similar family structures.

Family support, especially support from the husband, was the most recognised and desired support from participants in this research. During the interviews, it was noted that the family support experienced by the pregnant women also depended on their family socio-economic situation. For example, Participants 6 and 18, from higher socioeconomic backgrounds, shared their positive experiences of being physically and emotionally supported by their husbands and family members. According to the participants 6 and 18:

*My husband works in the office, I stay home, and my in-laws are old, so they stay at home. They tell me not to do any heavy work. They do help me as much as they can. Today, I am here for health check-ups with my mother-in-law. (P6, Urban)*

*I do feel having my husband with me is better. I share my concerns, happiness, emotions, and wishes with him. Women who do not have husbands with them must be experiencing distressful times. Women cannot share all their emotions with other people or with in-laws. (P18, Urban)*

The quotes above showed that women from better family socioeconomic backgrounds are relatively well supported. However, some women from lower socioeconomic backgrounds do not seem to enjoy the same level of support. This could be because of socioeconomic circumstances and cultural norms that is strictly practised in rural areas. For example, Participant 15, who lived in an extended family in a rural area, said:

*My husband goes out of the house for daily work. I feel and wish my family could support, care and love me more. My in-laws are okay. They are old people, they think differently. We live in a village. They have lived their whole life in this village. They do not know how things are these days. For example, health check-ups and taking rest during pregnancy. [……..]. I feel my husband’s love and care for me is okay, not too much, nor neglected. I wish I could get more support and care from my husband. I think men do not know what to do and how to look after pregnant women. Men are also busy at work, so they cannot care enough or give enough time to their pregnant wives. My husband never comes to this hospital. He says you go. Maybe he is not interested in coming to the hospital during the pregnancy health check-ups. This is a woman’s thing for him. (P15, Rural)*

The quote above by Participant 15 shows the deeply rooted patriarchal norms where pregnancy and maternity care are seen as ‘women’s thing’. If women wish to have their husbands’ support as primary support, then these cultural norms, alongside financial constraints and employment of their husbands, may act as a barrier that may put them at risk of poor mental health. Participant 7 stated that her husband’s jobs and experience of support during her pregnancy as:

*He (husband) went to Qatar for labour work after marriage. He went to work for the first time during my first pregnancy. I was 4 months pregnant at that time. He came back after 3.5 years. Then he stayed at home for about 1 year. Then again, he went to Qatar for 2 years. […….]. This time he came for a holiday for 2 months, and he was scheduled to go back. But due to the coronavirus, the lockdown started. I wish my husband is staying at home with me, but he does not have any work here (locally). What to do? We do not have any income sources. Otherwise, he would not go away for work during my pregnancy (P7, Rural)*

Despite having the desire to have support from their husbands during pregnancy, for women, it is not always possible, which may put them at risk of poor mental health. In certain circumstances, the women may experience violence and vulnerability. Participant 9, whose husband is working abroad, shared her experience as:

*I do not have anyone who can help me in this difficult time. I hope these nurses and this hospital will help me. I do not have any support in the society as well. I am worried. Because I do not know where to go, what to eat, how to survive, and how to raise this baby once born. My health is not good. My husband says the man who did the rape is responsible for my future. He (husband) says he is not responsible for looking after me and neither allows me to stay home. I have an illness as well. I don’t know what to do. I am worried so much. I don’t know where to go, and my family/relatives do not want to see me. I have nowhere to go. Family/relatives and society do not believe that this is a rape case. They think I am lying. They all think I am a bad woman. They think I am characterless. They talk about doing physical violence to me in the village. I do not have anyone who supports me. (P9, Rural)*

The above quote reflects the reality of women whose husbands are not with them during the pregnancy and are at risk of discrimination and abuse. Sometimes, women’s circumstances intersect to present compounded risks to them, such as financial circumstances, the absence of a husband, women’s empowerment, the negative attitude of society towards women, and the lack of the social security system in the country. Participant 13 lives with her husband, and therefore, feels more confident in receiving physical and psychological support when required. But she feels sorry for other women with reference to her experiences during pregnancy. She stated her experience as:

*Many women, my neighbours and relatives, do not have their husbands at home. Their husbands are working abroad or out of the village [……….]. Many families neglect women because they are daughters-in-law. In our society, the daughter-in-law’s position is the lowest while experiencing power within the family. Many women cannot even ask for help when they have leg swelling during pregnancy. They are hesitant to ask for help or to go for a health check-up. Women whose husbands are not with them are experiencing more problems. For example, if my husband is busy with his work, I still feel confident that if something goes wrong, my husband is with me and can come to me anytime I need. I feel so confident. I feel sad for those women who are alone without their husbands with them. (P13, Urban)*

From the experience of pregnant women, it is observed that the presence of a husband during pregnancy is important for physical, psychological and emotional support that is likely to improve the well-being of pregnant women and their pregnancy experience. However, many women do not receive adequate support from their husbands due to the sociocultural circumstances in which they live. Beyond husband support, women also seek the support of relatives and neighbours, which is presented below.

### Support from relatives, neighbours, and community

This section demonstrates how the mesosystem, within ecological theory, where relationships with family, relatives, and community members shape the support women receive during pregnancy, is influenced. An intersectional perspective helps explain that access to this support is not equal for all women, as it is influenced by factors such as education, where they live, and how their social roles are perceived, which can increase vulnerability for some women. Pregnant women recognise that the support of relatives and neighbours is crucial when they cannot rely on their immediate family, especially when travelling to health facilities for a health check-up and staying in town. For example, Participant 12, who lives in a rural area, said:

*I am staying at my relative’s place in this town. I came here 3 days ago and am still waiting for my delivery. I don’t know how long I have to wait for delivery. […….]. As my relatives are here in this town, it is easier for me to come here for better health services rather than going anywhere else where I do not know anybody. (P12, Rural)*

The women who lived in rural areas shared a similar story. They need support when they travel for health check-ups in town or to the village health facilities. However, they seek help only if they do not have anybody to support them in their immediate family. Participant 5 discussed the support she receives from her relatives:

*Regarding relatives’ help, if my family can’t come with me for health check-ups at the health post in my village, then we have relatives who help me. My sister-in-law takes me to the health post in the village. Other than that, I have not received any help from anybody. (P5, Rural)*

Neighbours and relatives have been helpful and supportive when women, especially those from rural and lower socioeconomic backgrounds, need to borrow money to manage costs related to pregnancy and delivery. Participant 7 shared her expectations from relatives and neighbours.

*I feel neighbours can help me, especially my relatives who will help me. In case of an emergency and we have to go to another hospital from this hospital, then we might need financial help from relatives and neighbours. I am managing myself so far. I wish people would show interest and advise me on how to do better during my pregnancy. I also wish for help to look after my baby and me after delivery in the community. At that time, I will be physically inactive for a few days. Relatives and family members from my parental side will come and stay with me for a few days and help me to go through the first few days after delivery. (P7, Rural)*

From the experience of Participant 7, finance and any form of support would be good for her. However, when exploring women’s experiences in urban areas, their perception of support from neighbours and relatives is different. For example, Participant 6, who lives in an urban area, shared her expectations of support, which did not include financial expectations. According to her:

*In the community, people ask me when my due date is. They advise me on what to do and what to expect. They ask me how everything is going. Our neighbours are good. They ask me often about how I am feeling. They advise me to eat healthy food, take rest, etc. Socially, I feel good. So far, I have not taken any help from people in the community. I am managing well. (P6, Urban)*

Participant 13 further highlighted how society perceives women in need. She expressed her positive experiences with neighbours and relatives but also said that the socioeconomic circumstances of women also matter as women from the better socioeconomic are more likely to receive better social support. According to her:

*In society, I have good relationships. Older women or previous pregnant women say good things to me. They say to eat healthily, take a rest, and go for a health check-up. I feel supported by them. I meet with other pregnant women too. We have relatives and neighbours who are pregnant. We ask each other how everything is. In the society, I also notice independent women are more supported in the family and society. I think the reason is women can explain and convince families what they wish to do. (P13, Urban)*

In this research, the women’s narrative sets a clear message that women from lower socioeconomic backgrounds have different expectations than women from better socioeconomic backgrounds in terms of the social support they expect during pregnancy. For example, Participant 15 shared her experience as such:

*Physically, it is difficult to be pregnant. I feel weak. Financially, we are a poor family. It is also a problem. In terms of support from others, I do not see any support from anybody. I do not talk about my pregnancy with anybody. In the community, people are busy working on their chores. I feel uncomfortable talking to them about my pregnancy. Society is ok for me. I do not feel anything special. In terms of support, I do not have any experience. I have not experienced any emotional or physical support from the people in the community. People say to take rest, how is it possible to take rest without working, who will do all the household work and look after cattle? I do not feel or expect any support in the village, but I wish people would help me if I need anything. (P15, Rural)*

Some women may feel less supported in the community in different sociocultural circumstances and feel socially isolated. For example, Participant 19 lives in a rural area and was pregnant at an old age, as per social understanding old age. She shared her experience in the community as:

*In the village area, people are talking behind my back. They think I am pregnant because I want to give birth to a baby boy. That is not true. I wish to have a girl again. Villagers say I am pregnant at this old age because I want a baby boy. People laugh at me. I do not feel very much welcome environment in the community and by my neighbours. But few women have supported me. It hurts me when people say I am pregnant at this old age just to give birth to a baby boy because I do not have any boys. (P19, Rural)*

From the narrative of women, it is highlighted that women receive and have different expectations from a society based on sociocultural circumstances; on the other hand, women from lower socioeconomic backgrounds may receive less support despite they are in great need of support. These inequalities create further vulnerability for those women who are really in need of support during pregnancy. Beyond the help from family, relatives, neighbours, and community, this research also explored if there is any organised community support for these women without discriminating based on sociocultural circumstances, which is presented below.

### Organised community support

It was important to explore if there were any organised social support or activities available for pregnant women within the community since not every pregnant woman had a positive experience with the informal support they received, as discussed above. The lack of organised community support points to a weakness in the exosystem, where community-level institutions are expected to provide assistance. An intersectional perspective reveals that women who are most in need are particularly those from rural areas, low-income households. In this research, the participants’ experience showed that none of the pregnant women from urban or rural areas were aware of any organised activities in the community. Participant 18, who lived in an urban area, stated:

*I have not met any other pregnant women in the community. We do not have any groups or organisations that gather pregnant women and provide information. There are other pregnant women in the area, but we are not very much connected to each other and do not have the environment to share experiences of our pregnancy. (P18, Urban)*

Women from rural areas revealed that financial concerns were one of the biggest challenges during their pregnancy, and they were expecting financial support as the most desired support from the community, rather than any other form of support. Participant 17 and Participant 4 expressed their financial concerns and possible support from the local community as:

*The community can help other pregnant women and me financially in case families of pregnant women are in a difficult position. They can lend some money. That is all the community can do for me. There is no guarantee who can support me in that time of need. We all women experience financial stress during pregnancy. We never know how much expenses can be during labour and delivery. It is very uncertain, and no one can be assured, and this uncertainty of support and expenses is a worry for me. (P17, Rural)*

*The village committee (municipality) said they will support me with some expenses later, once the baby is born, that is to support delivery-related expenses. (P4, Rural)*

In this research, it is identified that there is no organised and guaranteed financial support within the community for pregnant women. They rely on goodwill gestures from the members of the community, and some noted possible support from the village municipality, even though support could be provided after the delivery of the baby. That means no guaranteed support during the pregnancy is identified in this research based on the experience of pregnant women. Beyond community support, this research also explored whether there was NGO/INGO support available for pregnant women, which is presented next.

### NGO/INGO support

This theme highlights a significant gap at both the community (exosystem) and policy (macrosystem) levels, where Non-Governmental Organisations (NGOs) and International Non-Governmental Organisations (INGOs) could have provided essential support. The complete absence of such assistance in participants’ experience suggests that structural neglect, shaped by factors such as gender, rural location, and poverty, further disadvantages women who are already socially and economically marginalised. NGOs and INGOs in developing countries make a significant difference in improving the health and well-being of the marginalised community. In this research, pregnant women were asked if they received any NGO or INGO support during their pregnancy. The findings, based on the self-reported experience within the sample of participants in this study, suggest that no single woman received any support from NGOs/INGOs, nor were they aware of the available support from the NGOs/INGOs. For example, Participant 7 stated:

*I have not experienced any support from the society or NGOs/INGOs. I have not taken any help from family relatives as well. I am managing myself so far. I wish people are showing interest and advising me on how to do better during my pregnancy. I wish there is any other support other than health services, but I do not know how and who could provide that support. Do you know any organisations? (P7, Rural)*

The women in this research didn’t experience any support from the NGOs/INGOs. However, some women do recognise the importance of these organisations. For example, Participant 6 said:

*Many families do not have cash in their pockets. It is hard for those women to receive maternal health services. I feel if NGOs/INGOs could support needy women, then many lives would be saved. Even free ambulance service could be lifesaving. Many poor families cannot call for ambulance services for pregnant women, and these pregnant women suffer at home. (P6, Urban)*

In the interview with the pregnant women, they unanimously agreed that there is no INGOs/NGO support available for them. Beyond the support of NGOs/INGOs, the women raised concerns about the maternity leave policy, which is presented next.

### Maternity leave policy

This finding relates to the macrosystem, where government policies shape women’s everyday experiences. From an intersectional perspective, the limited maternity leave mainly impacts negatively on women working in informal or unstable jobs, making existing health inequalities worse. Only one woman was noted as an office-working woman in the interviews with pregnant women in this research. She raised concerns related to her job insecurity and the social injustice in maternity leave policy. She stated her concerns as:

*I am still waiting to apply for my maternity leave. We can take it before or after the delivery. We get only 90 days of paid maternity leave but that is not enough for us. This is the same amount of leave as for the government sector. I think a minimum of 6 months of maternity leave would be more practical. In a private company, women need to leave the job if they want more or longer leave for maternity reasons. We do not have any law that protects the job for women after maternity leave. For example, if I take a longer leave, then it is not guaranteed the company will allow me to return to my existing job. It is a significant job insecurity. For a mother, for a baby, and for a nuclear family, we must have a longer and reasonable maternity leave. At least breastfeeding is the most important right of my baby. My baby will not have exclusive breastfeeding if I return to work, which is my major concern now. I have a master’s in finance, and I am a working woman, but my future is very uncertain because I am not sure if I can return to work after the birth of the baby. The company policy does not guarantee a return to work after 3 months of maternity leave. [……….]. The company will recruit someone else. I am on probation period because the company does not want to employ permanent staff because of COVID-19 uncertainty. It is already an insecure job. So, job insecurity is very high among working mothers, in my experience. I have other friends who were pregnant before and they share the same experience. They could not spend enough time with their baby. We have to choose between the work or family life, which should not be the case. (P14, Urban)*

Participant 14 stated unmeasurable mental distress due to a lack of support from the national maternity policy. In addition to that, Job insecurity further concerning in the informal job market. Participant 18 left her job due to sickness related to her pregnancy. She shared her experience as such:

*I used to work in a tea factory in this district. I worked for 3 years for that factory. Now I have left the job because I am pregnant. I was on a temporary contract. Temporary contact is like a labour job. If I go to work, I get money. If I don’t go to the job, I do not get any money. There is no support for pregnant women. I started feeling sick after being pregnant. I used to vomit at that time. It was difficult for me to work. So I left that job. It has a financial impact on my family, but I have no choice and no support from the workplace. (P18, Urban)*

Participant 18 mentioned that despite working in the factory for three years, she was not supported to take sick leave or time off, and neither did she get any paid or unpaid maternity leave. The women’s experience raised serious concerns regarding support from the national maternity leave policy.

## Discussion

From the experience of pregnant women in this research, it is reported that social support could come in different forms and sources, and the support received by the pregnant women could enhance their pregnancy experience, confidence and sense of security. Following the concepts of ecological theory and intersectionality, this study presents an interpretation of the findings at different levels of support experienced by pregnant women and the challenges they face across multiple ecological levels.

### Support within the microsystem of the ecological theory

Looking at Bronfenbrenner’s ecological theory [26], a pregnant woman sits at the centre of the circle with her specific needs. It is noted that their husband, immediate family members, relatives and neighbours are recognised as the prime source of support during pregnancy, which can be placed in the microsystem of the ecological theory. The women in this research stated that their husbands’ support is the most important and desired support during pregnancy, and it is crucial. Still, other family members, such as in-laws, were also acknowledged for providing support during pregnancy. Although support from other family members was reported in the absence of the husband, the husband’s support during pregnancy had the potential to improve wellbeing of pregnant women, which is also acknowledged in previous research, conducted in Nepal, concluding the well-being of pregnant women could be improved if they are supported by their husband [42,43]. Pregnant women feel comfortable and confident sharing their concerns with their husbands and receive greater reassurance that promotes their mental well-being [44]. On the other hand, a lack of support from the father may cause adverse emotional and behavioural health outcomes for the mother and baby [45]. Although the women in this research agreed on the importance of husbands’ support, it was also noted that in many circumstances, the women may not receive adequate support from their husbands. For example, husbands migrate for employment opportunities. There is a sociocultural context, such as husbands working on daily wages and not being able to spend time with pregnant women, and sometimes cultural norms that pregnancy is a woman’s thing, which can be identified as a barrier to receiving support from their husbands during pregnancy. Here, we see multiple factors intersecting to present compounded barriers. In many cases, the intersection of the absence of a husband during pregnancy and their lower socioeconomic circumstances puts the women at an increased risk of poor mental health and well-being [22]. From the women’s narrative, it was noted that women are at risk of poor mental health if they experience poverty and lack of support from their husbands during their pregnancy, compared to those women whose husbands are with them at the time of pregnancy, despite the economic hardships. This means some women experience an intersection of multiple factors coming together that put them at risk of poor mental health compared to others [46].

In the interviews, it was observed that a woman’s relationship with family, neighbours, relatives, and the community also determines the support they receive during pregnancy, which is within the understanding of the mesosystem of the ecological theory. Findings also noted the intersection of social status that influences available social support for pregnant women, such as the women’s relationship within the family and neighbourhood, which is influenced by the socioeconomic status of the women. Our findings confirm the evidence from the previous research [47] that women from lower socioeconomic backgrounds are unlikely to receive the same level of support compared to women from higher socioeconomic backgrounds. Outside the family circle, a few women had the experience of informal social support in the community. The common support from relatives and neighbours was for information about the mother and baby’s health, which can be seen as a form of emotional and informational social support provided by neighbours [20]. However, the women from rural and lower socioeconomic backgrounds asserted that they needed tangible forms of support during the pregnancy, such as financial support or help with household chores.

### Support within the exosystem of the ecological theory

In this research, organised community support fits well in the exosystem of the ecological theory. In this research, the women understand there is no organised social support in the community, which means no organised support system exists within the community to promote the health and well-being of pregnant women living in that community. Generally, community organisations have an understanding of the needs, cultural norms, and socioeconomic circumstances of the local population. Around the world, community-level programmes seem to promote maternal mental health effectively [48]. For example, Mothers for Mothers [49], Young Mums Together [50], Mums and Babies [51] other programmes in the UK and Single Mom Programs in Canada [52] have been effective in supporting pregnant women and positively impacting the mental health of new mothers and pregnant women. At the community level in the context of low-resourced countries, women’s groups and volunteer peers have been successful in promoting maternal and child health in African regions [53]. The self-help community approach has seen significant success in promoting empowerment and sharing information about mental health within the group, and it has been more effective for those with lower social support in the United Kingdom [54]. Such initiatives could be an ideal community approach to promoting pregnant women in Nepal by providing social support and creating a channel for sharing maternal health-related information. Within the literature, AAMA SAMUHA, internationally known as Health Mother’s Group (HMG), already exists in Nepal [55], which has a similar principle to that of the concept of a self-help group [54]. In Nepal, the effective implementation of the Health Mother’s Group (HMG) could be a great source of organised social support so that women, particularly disadvantaged women, could benefit from the group to improve their health and well-being. However, the previous research noted that HMG had achieved very limited success as very few women were active users of the programme, and this was reflected in this research as none of the pregnant women in their interviews referred to associating themselves with this community-organised activity. However, previous research reported that there is a lack of community awareness regarding HMG meetings and benefits, a lack of motivation of Female Community Health Volunteers (FCHVs), inaccessible transport to reach the group to participate, including time and cost, and a heavy burden of the household commitments of the participant [56]. If these barriers are addressed, then it is likely that pregnant women can benefit from the existing HMG in the community.

Inadequate financial support was one of the major concerns for pregnant women from rural and lower socioeconomic backgrounds observed during the interviews. The support noted was promised after the delivery of a baby in a few rural municipalities on an irregular basis, and this support may not be available for women with unsuccessful deliveries, and there was no compulsory provision for all regions of the country. The financial provision of support was dependent on municipalities (local government), but there were no obligations or formal policies for municipalities to support pregnant women financially. The health of pregnant women can be promoted if there is an adequate welfare system that recognises the needs of people from disadvantaged communities through NGOs/INGOs, organised and structured forms of support [57,58]. None of the pregnant women during interviews recognised any support experience they had from the NGOs or INGOs, which is in line with previous literature [22]. Within the literature, it is well recognised that support from INGOs/NGOs significantly promotes health and well-being [59]. Despite having an extensive list of NGOs/INGOs in Nepal [60], it was somewhat surprising to see that not a single pregnant woman recognised any support from the organisations. To our knowledge, no previous study or publication has mentioned any organised or charity support for pregnant women in Nepal except the Health Mother’s Group, which was reported to have limited effectiveness. This gives us space to create arguments that pregnant women need community organisations that provide organised and guaranteed support and activities to promote their mental health and wellbeing in Nepal.

### Support within the macrosystem of the ecology theory

During the interviews, only one woman was working in the office in this research. Still, it is important to present the distress experienced by the working woman in this research, as there is an increasing number of women working in Nepal who may have similar concerns related to the maternity policy of the country. The maternity leave policy of Nepal sits within the macrosystem of ecological theory. A couple of women expressed their insecurity and financial concerns associated with the maternity policy of Nepal. Women expressed a lack of implementation of the policy in the private and informal working environments, while insufficient support from the policy can disadvantage working women in both formal and informal sectors. It was noted that the shorter length of maternity leave could significantly impact breastfeeding women and child development, and it is reasonable for women to be concerned due to this inadequately supported maternity leave policy in Nepal. Within the law, women get maternity leave for only fourteen weeks before or after the delivery and get full remuneration for only sixty days [61], which seems to be insufficient support for women to exclusively breastfeed and manage childcare. In addition, the pregnant woman in this research asserted a high level of job insecurity that potentially discourages women from working outside while enjoying maternity and family life. As girls’ education is increasing significantly in Nepal and many developing countries, it is likely that we will see more women in the workplace. Therefore, policies like maternity leave that support and protect them in the job market are invaluable and must be revised to address the needs of working women in the future. Other than the maternity leave policy, women in this research did not mention any other social security policies that provide them confidence and a sense of security while enjoying the maternity journey.

## Strength and limitations of the study

This research was conducted at a district hospital in the eastern region of Nepal, where pregnant women came to access antenatal health services. The experiences presented in this research may differ among women from different socioeconomic and cultural contexts of Nepal, which limits the generalisability of this research. However, we believe these findings provide a strong basis for future research and can be referenced to understand the role of social support in promoting the maternal health of pregnant women in similar sociocultural contexts in Nepal and other South Asian countries. As this research was conducted at a hospital, there could be potential participants with different experiences who could not come to the hospital. There could be women who wished to be part of this study but couldn’t be due to time limitations while completing their antenatal health checkup. However, we argue that purposive sampling allowed us to select the participants with diverse characteristics and possibly presented the voice of the majority of Nepalese pregnant women on the major issues raised by the women. A virtual interview may have discouraged women from discussing freely. In order to address the issues of trust, the researcher developed a good rapport and spent time with participants to build trust before starting the formal interviews, and we feel confident that there was no compromise in the quality of interviews conducted in this research. While we acknowledge that it is possible that being an insider the values and belief of the researcher may influence the interpretation of the data, we also argue that the supervisors of this research scrutinise the data analysis process where we feel confident that the researcher’s personal views had limited space to play in any role in data analyses and interpretation in this research.

## Conclusion and recommendations

This paper aimed to present pregnant women’s experience and understanding of social support in Nepal. It is noted that social support for pregnant women can be in different forms and from different sources, but it should promote the health and well-being of pregnant women. We conclude that social support can improve the well-being of the recipient. However, as seen in this research, not all pregnant women receive the same level of support in the family and community, which means inequalities in the health experiences of these pregnant women are likely due to unequal social support for the women. This paper concludes that the husband’s support is the most desired support by pregnant women, which is not always possible due to several socioeconomic and cultural factors in which the women live. We recommend creating awareness about the importance of husbands during pregnancy as a part of antenatal education to ensure women’s well-being. We also conclude that women experience limited or no social support due to the discriminatory attitude of society towards women from lower socioeconomic backgrounds, while the absence of organised social support means that women rely on the goodwill gestures of people in the neighbourhood and have nowhere to go for much-needed guaranteed support during their pregnancy. Therefore, we strongly recommend that an organised form of support be available in the community for pregnant women. This research also acknowledges that the women experience no organised NGO/INGO activities during the pregnancy, but we have noted AAMA SAMUHA (Health Mother’s Group) within the literature. We believe addressing the issues of AAMA SAMUHA and implementing them effectively would make a positive impact on pregnant women, as it would promote social support in the community for these women. The role of the municipality in promoting maternal health was recognised by women in this research, which is not guaranteed in the policy. We recommend that all the municipalities in Nepal provide guaranteed support to pregnant women, especially women from lower socioeconomic backgrounds. Based on the experience of the women, we conclude that the maternity leave policy is inadequate and ineffective, while the implementation of the policy in the informal job market is unnoticeable, which may put working pregnant women at risk of vulnerability. This research recognises that this policy in Nepal should be effective in promoting women’s employment opportunities, maternity allowance and protecting maternity rights. We recommend that women’s voices be considered in future maternity policy revision and implementation.

## Supporting information

S1 File Interview schedule. (DOCX)

S1 Table Demographic characteristics of participants. (DOCX)
