# Supplementary material for: Understanding social support during pregnancy: A qualitative study of women’s lived experiences in Nepal
Source: PLoS One. 2025 Oct 17;20(10):e0333885. doi: 10.1371/journal.pone.0333885 (PMC12533895; doi:10.1371/journal.pone.0333885)
Supplement: S1 File — (DOCX) [file pone.0333885.s001.docx]

# Interview Schedule

In both the interviews and surveys of this research, it would will collect some demographic information. Use of demographic information will provide precise and detail information about the population groups who are most at need of the required resources and support. In the long run, the information will be used to understand and explore further the mental health and wellbeing of the particular population group and to health resource allocation.

**In-depth interviews with Pregnant Women**

***Opening statement***

Thank you very much for agreeing to participate in this interview. As you are a pregnant woman, it would be a good idea to interview you, so that I can have more information from your perspectives.

I would like to ask you some questions about your experiences you have had about mental health during your pregnancy. I hope to use this information in my research to provide clarity and better understanding about mental health issues pregnant women are facing. The interview should take about 30 to 60 minutes. Are you available to respond to some questions at this time?

- **Transition:** Let me begin by asking you some questions about where you do you live

1. Please tell me a story of your normal one day

**Prob:**

- How do you spend your day?
- What do you do?
- What is the hardest thing to do?
- Do you like to eat very often in this pregnancy?

1. Can you please tell me what is your experience of being a pregnant woman?

**Prob:**

- Is this your first pregnancy?
- How are you feeling about your pregnancy?
- Are you enjoying your pregnancy?
- Is there anything you are struggling for?
- Do you think pregnancy is important for women?

1. What are the supports available for you?

**Prob:**

- How often do you need supports?
- What type of supports are you getting?
- Are you well supported in your family/community?
- Are there any social supports available to you?
- Do you often meet other pregnant women?

1. How is your experience receiving health care services?

**Prob:**

- Are you happy to receive the health services available to you?
- How do you feel your day in the hospital?
- Do you think the services you are receiving is adequate?
- What could be done to provide better support and care to her?
- Are there any difficulties or barriers that discoursing you to receive the health services?
- Can you tell me your opinion about Safe Motherhood Programme?

***Closing Statement***

You were very involved talking about your experiences. I appreciate your time and consideration.

- Transition: Well, it has been a pleasure talking to you

**Final Question:** Is there anything else you think would be helpful for me to know?

Thank you again for your valuable time for this interview.
