# Supplementary material for: Understanding social support during pregnancy: A qualitative study of women’s lived experiences in Nepal
Source: PLoS One. 2025 Oct 17;20(10):e0333885. doi: 10.1371/journal.pone.0333885 (PMC12533895; doi:10.1371/journal.pone.0333885)
Supplement: S1 Table — (DOCX) [file pone.0333885.s002.docx]

## Demographic Characteristics of Participants

Table 1: Demographic Characteristics of Pregnant Women (In-depth Interviews)

| **Participants** | **Age** | **Age at marriage** | **First pregnancy**  **Yes/no** | **Age of first pregnancy** | **Number of pregnancies** | **Stage of**  **current pregnancy/**  **trimester** | **Planned pregnancy**  **Yes/no** | **Education *** | **Employment** | **Husband Employment** | **Living arrangement (extended/**  **nuclear family) **** | **Place of residence** | **Distance to district-level health facility** |
| --- | --- | --- | --- | --- | --- | --- | --- | --- | --- | --- | --- | --- | --- |
| P1 | 24 | 21 | Yes | 24 | 1 | 2^nd^ | No | Undergraduate | No | Business but currently impacted by covid-19 | Extended | Rural | More than 1 hour |
| P2 | 18 | 14 | No | 14 | 2 | 3^rd^ | Yes | Literate | No | Labour job daily basis | Extended | Rural | Less than 30 minutes |
| P3 | 21 | 19 | Yes | 21 | 1 | Post-dated | Yes | Intermediate | No | Bus driver | Extended | Rural | More than 1 hour |
| P4 | 20 | 18 | Yes | 20 | 1 | 3^rd^ | Yes | Primary | No | Self-employed – contract job | Extended | Rural | More than 1 hour |
| P5 | 30 | 17 | No | 18 | 2 | 3^rd^ | Yes | Lower secondary | No | Farmer | Extended | Rural | More than 1 hour |
| P6 | 30 | 25 | Yes | 30 | 1 | 3^rd^ | Yes | Graduate | No/Past teacher | office worker | Extended | Urban | Less than 30 minutes |
| P7 | 24 | 16 | No | 17 | 2 | 3^rd^ | No | lower secondary school | No | Work in labour market abroad | Nuclear | Rural | More than 1 hour |
| P8 | 20 | 19 | Yes | 20 | 1 | 3^rd^ | No | intermediate | No | Works in bank | Extended | Urban | Less than 30 minutes |
| P9 | 45 | 21 | No | 27 | 6 | 3^rd^ | No | Literate | No | Works abroad in the labour market | Nuclear | Rural | More than 1 hour |
| P10 | 33 | 32 | Yes | 33 | 1 | 3^rd^ | Yes | Upper secondary | No | Farmer | Extended | Rural | More than 1 hour |
| P11 | 40 | 21 | No | 23 | 3 | 3^rd^ | No | Literate | No | Farmer, live sticks | Nuclear | Rural | More than 1 hour |
| P12 | 28 | 22 | No | 23 | 2 | 3^rd^ | No | intermediate | No | Farmer, live stocks | Extended | Rural | More than 1 hour |
| P13 | 28 | 21 | No | 24 | 2 | 3^rd^ | Yes | intermediate | Self-employed -small grocery shop | Vehicle business | Nuclear | Urban | Less than 30 minutes |
| P14 | 28 | 27 | Yes | 28 | 1 | 3^rd^ | Yes | Postgraduate | office worker | Office worker | Nuclear | Urban | Less than 30 minutes |
| P15 | 38 | 37 | Yes | 38 | 1 | 3^rd^ | Yes | Literate | No | Carpenter | Extended | Rural | 30 minutes to 1 hr |
| P16 | 24 | 16 | Yes | 24 | 1 | 3^rd^ | Yes | SLC | No | Tea farmer | Nuclear | Rural | More than 1 hour |
| P17 | 27 | 20 | No | 20 | 2 | 1^st^ | No | Upper secondary | Self-employed | Farmer | Nuclear | Rural | Less than 30 minutes |
| P18 | 25 | 18 | No | 19 | 2 | 2^nd^ | Yes | Literate | No | Bus driver | Extended | Urban | Less than 30 minutes |
| P19 | 42 | 27 | No | 28 | 4 | 2^nd^ | No | Intermediate | No | Farmer | Nuclear | Rural | More than 1 hour |
| P20 | 31 | 13 | No | 16 | 4 | 2^nd^ | No | Literate | No | Carpenter | Nuclear | Urban | Less than 30 minutes |

* Educational Terminology used in the table above is in line with the Census 2021 Nepal

<https://censusnepal.cbs.gov.np/results/files/result-folder/National%20Report_English.pdf>

**Nuclear family: Couple living with children under the same roof; Extended family: Couple living with children and grandparents under the same roof.

**Summary of the participants:**

1. 14 women resided in rural areas
2. 9 participants stated the current pregnancy was unplanned
3. 9 participants were 1st time pregnant
4. 8 women had the experience of first pregnancy at the age of 20 years or below
5. 5 women married at aged 20 and under
6. 1 woman second time married
7. 8 women live in their nuclear family
8. 6 women – no formal education but able to read and write but literate by definition of the 2021 census of Nepal
9. Only 3 women employed (1 working as in a private sector, office job, 1 woman – selling fruits, veg, and milk in the town, and 1 woman – small grocery shop in a room in the front room of the house where she lives)
